# Does the disclosure of key audit matters improve the audit quality for sustainable development: Empirical evidence from China

**DOI:** 10.1371/journal.pone.0285340

**Published:** 2023-05-04

**Authors:** Jintian Lin

**Affiliations:** School of Accounting, Fujian Jiangxia University, Fuzhou, China; University of Baltistan, PAKISTAN

## Abstract

In this paper, taking the 14837 annual audit reports of 4159 listed companies in Shanghai and Shenzhen Stock Exchanges from 2017 to 2020 as samples, and taking the information entropy value of KAM disclosures and the type of audit opinion as the agent indexes of the explanatory variable and the interpreted variable respectively, whether the disclosure of KAMs could improve audit quality had been empirically tested and analyzed. The results shown that: (1) The regression coefficient of information entropy value of KAMs disclosure was 0.1785, which shown a significant positive correlation, and was established at the level of 1% significance, that was to say, KAMs disclosure can improve audit quality. (2) The marginal effect coefficient was only 0.0081, which meant that there was some information redundancy in the KAMs disclosure and the enhancement effect on audit quality was weak. (3) In the robustness test, the interpreted variable was respectively replaced by audit cost (taking the natural logarithm of audit cost) and manipulated accrual profit (taking the absolute value of manipulated accrual profit), the regression coefficients of information entropy of KAMs were 0.0852 and 0.0017 respectively, which shown a significant positive correlation and were consistent with the results of the main regression test. (4) Further research shown that whether the industry in which the audited company was located and whether the audit institution was one of the international Big Four would affect the disclosure of KAMs, and then affect the audit quality in the same direction. The implementation effect of the new audit reporting standards was supported by these test evidences.

## 1 Introduction

In 2016, the new audit report reform requires KAM (key audit matter) disclosure, which aims to further enhance the information content of audit reports and ease capital market information asymmetry [1]. At the same time, it helps to improve the value of audit report in the fields of credit decision-making and capital market supervision [2, 3]. Relevant research shows that when the litigation risk of listed companies is high [4], the degree of digitization is high [5], or there are signs of fraud [6], auditors will strengthen the communication with the management of information, and then there will be more kinds of KAM disclosure, more content of risk topics and differences, in order to lower the text similarity of KAM to avoid audit risk. In addition, media reports can improve the information content of audit reports by influencing auditors’ KAM disclosure, and the original intention of audit report reform is realized [7].

However, most of the KAM disclosure has the sameness in practice [8]. Firstly, the types of KAM disclosures are mainly income recognition and asset impairment, which is not conducive to comprehensively reflecting the production and operation situation of companies and the development trend of the industry, and has limited results in enhancing the communication value of audit reports. Secondly, the number of KAM disclosures is relatively small, and the information value provided for the potential users of annual financial statements and audit reports may be limited [9]. Moreover, the content description and audit countermeasures of KAMs show the signs of routinization and stereotype, which may further reduce the information value of KAM disclosures. Research by Rongbing Huang and Zhang points out that there is imitative isomorphism in KAM disclosure, and imitating leads to boilerplate disclosure, which releases more information at industry level, but reduces information on firm characteristics [10].

If KAM disclosures are homogenized, namely following the same pattern or a mere formality, and are not corrected and guided in time, then the strength and effectiveness of the decision support of KAMs will undoubtedly be greatly reduced, which will also deviate from the original intention of the audit report reform. Therefore, whether KAM disclosures really enhance the personalization, readability and practicality of audit reports, improve the information communication and decision support value of audit reports, and promote the improvement of audit quality are one of the key issues in current audit empirical research. However, most of the existing studies only use a single year, or a single industry sample of listed companies for empirical analysis, or use case analysis, it is not able to demonstrate the impact of KAM disclosure on audit quality. Therefore, this paper empirically tests and analyzes whether the KAM disclosure can improve audit quality based on cross-year, industry-wide and large sample of listed companies.

Compared with previous studies, the possible contribution of this paper is as follows. Taking the 2017–2020 annual audit reports of listed companies in Shanghai and Shenzhen Stock Exchanges as samples and the information entropy value of KAM disclosures as a proxy indicator, in this paper the impact of KAM disclosures on audit quality was tested, which provides new proxy indicator ideas compared with the binary assignment proxy indicator in previous studies [11–13]. In addition, the impact of KAM disclosures in different sectors, industries and accounting firms on audit quality was tested, which makes the conclusion of this paper more comprehensive and reliable. Also, the implementation consequences of the new audit report standards were tested from the perspective of the information entropy value of audit reports, which provides experimental evidence for the standard-setting institutions and accounting firms to improve KAM disclosures.

## 2 Theoretical analysis and research hypothesis

### 2.1 KAM disclosure and audit quality

According to the new audit report standards, CPAs and accounting firms shall disclose KAMs when issuing the annual audit report of listed companies, including describing the KAMs and the reasons identified as KAMs, and audit countermeasures for KAMs, and not giving separate opinions on KAMs [14, 15]. It is the original intention to close the audit expectation gap between the information supply and demand parties, break the “black box” of audit information, improve the transparency of audit work, and improve the audit quality by means of the effect of market magnifying glass [16]. However, the audit expectation gap is absolutely impossible to be eliminated. The reasons are as follows. The public’s demand for information is unlimited, while the information that CPAs can provide is limited; the public’s requirements for practice quality are flawless, while the practice subjects, procedures and methods of audit have inherent limitations; the economic activities in the market economy are dynamically changing, while the audit standards give consideration to the quality and efficiency, cost and benefit. Therefore, CPAs’ decision of how to disclose KAMs is the result of weighing the advantages and disadvantages of rights and responsibilities and comprehensively considering the factors.

In China’s audit practice, CPAs and accounting firms are not required to separately express clear audit opinions on KAM disclosures, and only a few audit reports actively make conclusive comments on KAM disclosures [17]. Thus, the process of report users reading KAM paragraphs is essentially a process of information self-extraction and self-analysis, rather than a process of directly extracting conclusions. For this reason, their professional level and ability to analyze and understand are particularly important. Professional and high-level report users can get more valuable information when reading and using KAMs, so that they can make the best economic decisions, which reduces the possible risk liability of CPAs; unprofessional and low-level report users may obtain invalid information or even biased understanding when reading and using KAMs, so that they can make wrong economic decisions, which indirectly increases the possible risk liability of CPAs. However, CPAs disclose KAMs and issue audit reports before report users read and use KAMs, and they cannot control the scope, scale, level, purpose and demand of report users, so they must make decisions on KAM disclosures in advance according to professional judgment.

According to John von Neumann’s Game Theory [18], we assume that CPAs are completely rational and in pursuit of self-interest maximization, and form correct beliefs and expectations about the decision environment conditions and the behavior of other participants [19], and have enough practice level and competitiveness to freely choose high-quality or conservative KAM disclosure strategies. In an audit, when the CPA believes by professional judgment that disclosing more information may expose them to greater risks so that they may assume more responsibility, then he or she will choose conservative KAM disclosure strategy to reduce the quality of audit reports; when the CPA believes by professional judgment that disclosing more information may make he or she enjoy the benefits of reducing investor’s perception of auditor responsibility, especially when KAMs are consistent with major misstatement [20], then he or she will choose high-quality KAM disclosure strategy to improve the quality of audit reports.

On the one hand, just as it is impossible to be absolutely independent, CPAs cannot be completely rational. At the same time, limited by their practice level and competitiveness as well as various other factors, it is impossible to switch strategies freely and clearly when making decisions on KAM disclosures. So CPAs’ every communication and disclosure of KAMs will affect the audit quality [21] and audit risk [22]. However, different CPAs’ judgment and cognition of the disclosure and risk responsibility of KAMs are inevitably different, and the whole audit market is composed of many CPAs. Thus, it is, in theory, far more complex and changeable to investigate whether KAM disclosures improve audit quality from the audit market level than from the individual CPA level. On the other hand, in information theory, information is defined as a relevant concept of uncertainty, that is, the more dispersed the probability distribution, the greater the uncertainty, the higher the amount of information; otherwise, the lower the amount of information. Obviously, the amount of information from KPA disclosures is not necessarily proportional to the number of KAM disclosures, but is related to the probability distribution of KAMs. In practice, there may be the following situations. The number of KAM disclosures in a system is large, but there is information redundancy or high value information; the number of KAM disclosures in a system is small, but there is high value information or information redundancy. The research now has not confirmed the actual probability distribution of KAM disclosures in the audit reports of listed companies. Based on the above theoretical analysis, we proposed the competitive hypotheses *H*_1*a*_ and *H*_1*b*_.

*H*_1*a*_: KAM disclosures can improve audit quality.

*H*_1*b*_: KAM disclosures cannot improve audit quality.

### 2.2 Audit market concentration and audit quality

There are three representative views regarding the relationship between audit market concentration and audit quality, which is little studied. The first view is that there is a positive correlation between audit market concentration and audit quality [23, 24], that is, the higher the audit market concentration, the higher the audit quality; the lower the audit market concentration, the lower the audit quality. The second view is that there is a negative correlation between audit market concentration and audit quality [25], that is, the higher the audit market concentration, the lower the audit quality; the lower the audit market concentration, the higher the audit quality. The third view is that there is an inverted “U-shaped” relationship between audit market concentration and audit quality [26], that is, the audit quality based on the scale economies effect will gradually improve with the increase of audit market concentration. However, it will decline when it reaches a certain vertex, which is explained from the characteristics of the monopoly market, that is, when an industry is the monopoly structure, the dominator’s quality requirements may decline due to few or no competitors in the same industry. Obviously, there is no consensus on the relationship between audit market concentration and audit quality, which has a certain relationship with the different research design such as sample scope and size, selection of alternative variables, and validation of model construction. Most of the existing studies of the relationship between audit market concentration and audit quality put forward the hypothesis based on the logical relationship between accounting firm size and audit quality from the perspective of the principles of managerial economics such as industrial economic theory and enterprise organization behavior. This paper explores the relationship between audit market concentration and audit quality from the logical relationship between KAM disclosures and audit quality, because the audit market concentration will affect KAM disclosures.

First, when the audit market concentration is high, the head accounting firm may have high practice confidence and requirements in KAM disclosures based on its own strength and reputation, which may lead to two possibilities. One is “empirical” KAM disclosure due to blind confidence, and the other is “elitist” KAM disclosure that maintains a high professional level. At this time, small and medium-sized accounting firms are likely to have a “herd effect”, namely, to improve their own practice level and competitiveness by imitating the KAM disclosures of the head accounting firm. Obviously, as a role model, the development direction of the head accounting firm itself not only affects the majority of small and medium-sized accounting firms, but also ultimately affects the market direction of the whole audit market. Second, if the audit market concentration is low, there will be sufficient market competition. Under this type of market structure, accounting firms often have the typical characteristics that they are small, scattered and weak, and there is basically no big difference in practice scale and strength, which is not conducive to the growth of the industry in the long run. It may lead to two possibilities. One is the virtuous cycle of competition, where various accounting firms, in order to establish advantages in the fierce competition, constantly improve their competitiveness and practice level, and take high-quality audit service measures such as high-quality KAM disclosures to establish a professional image and attract target customers, so as to promote the steady improvement of the overall audit quality of the audit market. The other is excessive or severe competition, where a few accounting firms, in order to survive in the fierce competition, deliberately reduce audit fees and maliciously arrogate business as their competitive advantage despite the professional ethics, which results in the phenomenon of “Bad Money Drives out Good Money”, making the audit market into “The Market for Lemons”. As stated earlier, China’s current audit market is the competitive structure, still far from the monopoly structure. Thus, even if there is an inverted “U-shaped” relationship between audit market concentration and audit quality, China did not reach the peak of the “U-shaped” curve. So there is an undoubtedly monotony relationship between audit market concentration and audit quality in China. Based on the above theoretical analysis, we proposed the competitive hypotheses *H*_2*a*_ and *H*_2*b*_.

*H*_2*a*_: There is a positive correlation between audit market concentration and audit quality, that is, the higher the audit market concentration, the higher the audit quality; the lower the audit market concentration, the lower the audit quality.

*H*_2*b*_: There is a negative correlation between audit market concentration and audit quality, that is, the higher the audit market concentration, the lower the audit quality; the lower the audit market concentration, the higher the audit quality.

## 3 Research design

### 3.1 Research model and experimental design

In order to test the impact of communicating KAM disclosures on audit quality, we established the following Models (1) and (2). The audit market concentration of Model (1) was calculated using the business income of accounting firms, and that of Model (2) was calculated using the number of CPAs of accounting firms:

$$\begin{array}{l} AQ_{i,t}=\alpha_{0}+\alpha_{1}H{\left(X\right)}_{i,t}+\alpha_{2}CR_{4-Income_{i,t}}+\alpha_{3}CR_{8-Income_{i,t}}+\alpha_{4}HHI_{Income_{i,t}}\\ +\alpha_{5}Income+\alpha_{6}CPA+\sum Ind+\sum Year+\varepsilon \end{array}$$ (1)

$$\begin{array}{l} AQ_{i,t}=\beta_{0}+\beta_{1}H{\left(X\right)}_{i,t}+\beta_{2}CR_{4-CPA_{i,t}}+\beta_{3}CR_{8-CPA_{i,t}}+\beta_{4}HHI_{CPA_{i,t}}\\ +\beta_{5}Income+\beta_{6}CPA+\sum Ind+\sum Year+\varepsilon \end{array}$$ (2)

#### (1) Explained variable

The explained variable was the audit quality (*AQ*), with non-standard audit opinion as its proxy indicator. If a CPA issues a non-standard unqualified audit report, it indicates that he or she is independent and cautious in practice and gives high-quality audit opinions. At the same time, the information entropy value of non-standard unqualified audit reports is higher than that of standard unqualified audit reports. Therefore, although not all audit reports with non-standard unqualified opinions are high-quality audit reports, they can partly act as a proxy indicator for audit quality [27]. The value of standard unqualified opinion was set to 0, and the value of non-standard unqualified opinion 1.

#### (2) Explanatory variable

The explanatory variable was the KAM disclosures, with information entropy value (*H*(*X*)) as its proxy indicator. The information entropy value of KAM disclosures in each audit report was calculated according to Eq (1). And it was expanded by 1000 times due to the large gap between the information entropy value of KAM disclosures in a single audit report and other variables. If the regression coefficients *α*_1_ and *β*_1_ were significantly positive, then *H*_1*a*_ held true; otherwise, *H*_1*b*_ held true.


$$H(X)=-\frac{T}{Q}\ln\prod_{i=1}^{T}P(X_{i})$$ (3)

Where: *H*(*X*) was the information entropy value of a single audit report;

*T* was the number of KAM disclosures in a single audit report;

*Q* was the total number of KAM disclosures in all audit reports within a system;

*P*(*X*_*i*_) was the frequency of KAM *X*_*i*_ disclosures in the system.

#### (3) Control variable

Referring to the research of Li Yingmei et al., the annual business income and the number of CPAs of accounting firms were selected as control variables. Both will have an impact on the scale of accounting firms, which may affect the concentration and structure type of the audit market. Therefore, the two variables with the greatest impact on the scale of accounting firms were selected as control variables and included in the model for testing and analysis. If the regression coefficients *α*_2_, *α*_3_, *α*_4_, *β*_2_, *β*_3_, *β*_4_ were significantly positive, then *H*_2*a*_ held true; otherwise, *H*_2*b*_ held true (Table 1).

**Table 1:** Variable definitions.

Variable type	Variable name	Variable symbol	Variable definition
Explained variable	Audit quality	*AQ*	The value of standard unqualified opinion was set to 0, and the value of non-standard unqualified opinion 1
Explanatory variable	Information entropy value of KAM disclosures	*H*(*X*)	Information entropy value, that is, the logarithm of the continuous product of the frequency of KAM disclosures in a single report was corrected according to the ratio of the number of KAM disclosures to the total number of KAM disclosures, and then expanded by 1000 times
Control variable	Audit market concentration	*CR*_4−*Income*_	The ratio of the sum of the business income of top four accounting firms to the total business income of the industry
		*CR*_8−*Income*_	The ratio of the sum of the business income of top eight accounting firms to the total business income of the industry
		*HHI*_*Income*_	HHI based on the business income of accounting firms
		*CR*_4−*CPA*_	The ratio of the sum of the number of CPAs of top four accounting firms to the total number of CPAs of the industry
		*CR*_8−*CPA*_	The ratio of the sum of the number of CPAs of top eight accounting firms to the total number of CPAs of the industry
		*HHI*_*CPA*_	HHI based on the number of CPAs of accounting firms
	Business income of accounting firms	*Income*	The natural logarithm of the annual business income of accounting firms
	Number of CPAs of accounting firms	*CPA*	The natural logarithm of the number of CPAs of accounting firms
	Industry	*Ind*	The Guidance on the Industry Classification sets the industry dummy variable to control the industry effect
	Year	*Year*	Set the annual dummy variable to control the annual effect

### 3.2 Research samples and data sources

In this paper, the 2017–2020 annual audit reports of listed companies in Shanghai and Shenzhen Stock Exchanges, including the Shanghai mainboard, Shenzhen mainboard, GEM, and STAR Market, were collected. The samples collected were processed as follows: (1) excluding the audit reports of listed companies that are issued and cannot express their opinions; (2) excluding the audit reports of listed companies that have not been explicitly disclosed as KAMs; (3) excluding the audit reports of listed companies with incomplete information. This paper yielded 14,837 audit reports from 4,159 listed companies. The data of the listed companies and their audit reports was from the cninfo, the official website of the Shanghai and Shenzhen Stock Exchanges, Wind Information, CNRDS, and CSMAR, and the data of the accounting firms was from the official website of the CICPA. We read and recorded the name of the listed companies, the accounting period, the type of audit opinions, the accounting firms, the number of KAMs, and the specific content of KAMs, and determined the sector of the listed companies, the industry classification of the CSRC, etc. In order to eliminate the existence of outliers and extreme values in the data and ensure the authenticity and reliability of the research results, the continuous data in the sample was winsorized at level 1% to eliminate the influence of extreme values.

Referring to the division of KAMs by Liu and Dong [28], based on the latest KAM disclosures and the needs of this paper, the KAMs were divided into 30 categories and 66 items, which were not listed in detail due to the limited space.

## 4 Empirical results and analysis

### 4.1 Descriptive statistical analysis

Table 2 describes the descriptive statistics for each main variable. As the *AQ* and *H*(*X*) were the explained variable and explanatory variable of this paper respectively, they were analyzed in detail. According to the table, the minimum of *AQ* was 0, the maximum was 1, the mean was 0.049, and the standard deviation was 0.215, indicating that the number of audit reports with non-standard unqualified opinions only accounted for about 4.9% of the total number of audit reports. The minimum of *H*(*X*) was 0.114, the maximum was 24.383, the mean was 1.562, the standard deviation was 1.583, and the standard deviation was large, with large fluctuations and significant changes in the year, indicating that the entropy difference of KAM disclosures between the audit reports was large and it may have an impact on audit quality. Among the control variables, the standard deviation of *CR*_4_, *CR*_8_, *Income* and *CPA* was small, with small fluctuations. The standard deviation of *HHI*, especially the *HHI* based on the business income of accounting firms, was large.

**Table 2:** Descriptive statistical analysis results of main variables.

Variables	Obs	Mean	Std. Dev.	Min	Max
AQ	14837	0.049	0.215	0	1
H(X)	14837	1.562	1.583	0.114	24.383
CR_4-Income_	14837	0.338	0.01	0.324	0.35
CR_8-Income_	14837	0.537	0.017	0.518	0.562
HHI_Income_	14837	473.555	15.467	455.069	498.174
CR_4-CPA_	14837	0.212	0.009	0.201	0.226
CR_8-CPA_	14837	0.362	0.005	0.356	0.369
HHI_CPA_	14837	277.186	4.615	270.757	283.163
Income	14837	11.943	0.89	8.168	13.324
CPA	14837	6.989	0.668	2.398	7.808

### 4.2 Correlation analysis

Table 3 shows the Pearson correlation coefficients between the main variables to investigate whether the panel model had multicollinearity. According to the test results of Pearson correlation coefficient, except for the high correlation between *CR*_4_ and *CR*_8_, the correlation coefficient between the other variables was mostly below 0.8 [29], indicating that the panel model did not have a serious multicollinearity problem.

**Table 3:** Test results of correlation between main variables.

Variables	AQ	H(X)	CR4_Income_	CR8_Income_	HHI_Income_	CR4_CPA_	CR8_CPA_	HHI_CPA_	Income	CPA
AQ	1.000									
H(X)	0.057*	1.000								
CR_4-Income_	-0.027*	0.120*	1.000							
CR_8-Income_	-0.039*	0.125*	0.903*	1.000						
HHI_Income_	-0.038*	0.121*	0.939*	0.979*	1.000					
CR_4-CPA_	-0.044*	0.101*	0.593*	0.880*	0.811*	1.000				
CR_8-CPA_	-0.017	-0.049*	-0.509*	-0.190*	-0.192*	0.239*	1.000			
HHI_CPA_	-0.039*	0.029*	0.042*	0.411*	0.383*	0.755*	0.814*	1.000		
Income	-0.065*	0.037*	-0.113*	-0.107*	-0.106*	-0.075*	0.062*	-0.005	1.000	
CPA	-0.050*	0.000	-0.053*	-0.046*	-0.047*	-0.028*	0.035*	0.005	0.917*	1.000

Note: ***, ** and * were significant at the 1%, 5%, and 10% confidence levels, respectively, the same below.

### 4.3 Regression analysis

According to the principles of econometrics, the Models (1) and (2) were tested by Logit regression. The regression results are shown in Table 4. With the industry and the year remaining constant, the empirical results of Table 4 showed that the regression coefficient of *H*(*X*) against *AQ* was 0.1785, a significant positive correlation, and this result held true at the 1% significance level. It suggested that *H*_1*a*_ held true, that is, KAM disclosures can improve audit quality. The results of marginal effect showed that for each addition of *H*(*X*) unit, the probability of standard unqualified opinion with a value of 1 increased by 0.0081, indicating that the quality of KAM disclosures was not very high, with a certain information redundancy, and the effect on improving audit quality was weak.

**Table 4:** Results of main regression test of Logit model.

Variables	Model (1)		Model (2)	
	Regression coefficient	Marginal effect	Regression coefficient	Marginal effect
H(X)	0.1785***(8.52)	0.0081***(8.36)	0.1785***(8.52)	0.0081***(8.36)
CR_4-Income_	31.7886**(2.74)	1.4496**(2.73)		
CR_8-Income_	-6.0671(-0.53)	-0.2767(-0.53)		
HHI_Income_	-0.0328*(-2.01)	-0.0015*(-2.00)		
CR_4-CPA_			58.6862(1.60)	2.6762(1.60)
CR_8-CPA_			190.5711**(2.62)	8.6904**(2.62)
HHI_CPA_			-0.3111**(-2.54)	-0.0142**(-2.53)
Income	-0.8142***(-6.47)	-0.0371***(-6.34)	-0.8142***(-6.47)	-0.0371***(-6.34)
CPA	0.6708***(3.78)	0.0306***(3.75)	0.6708***(3.78)	0.0306***(3.75)
Cons	Yes		yes	
Industry	Yes		yes	
Year	Yes		yes	
Wald chi2	260.91		260.91	
Wald chi2 p	0.0000		0.0000	
Pseudo R2	0.0412		0.0412	
obs	14797		14797	

Comparisons of the impact of audit market concentration on audit quality showed that only the impact of *CR*_8−*Income*_ and *CR*_4−*CPA*_ on audit quality failed the significance test. Among them, the regression coefficient of *CR*_4−*Income*_ and *CR*_8−*CPA*_ against *AQ* was 31.7886 and 190.5711, respectively, a significant positive correlation, and this result held true at the 5% significance level; the regression coefficient of *HHI*_*Income*_ and *HHI*_*CPA*_ against *AQ* was -0.0328 and -0.3111, respectively, a significant negative correlation, and this result held true at the 10% and 5% significance levels. It fully showed that if the *CR*_4_ and *CR*_8_ were used as a proxy indicator of audit market concentration, then *H*_2*a*_ held true, that is, the higher the audit market concentration, the higher the audit quality; the lower the audit market concentration, the lower the audit quality. If the HHI was used as a proxy indicator of audit market concentration, then *H*_2*b*_ held true, that is, the higher the audit market concentration, the lower the audit quality; the lower the audit market concentration, the higher the audit quality. This may be related to the attributes of audit market concentration and HHI, which reflect the influence of head accounting firms and small and medium-sized accounting firms, respectively. It proved once again that the relationship between audit market concentration and audit quality was complicated, and different selection of proxy indicators may produce different results.

In addition, there was a negative correlation between *Income* and *AQ*, and a positive correlation between *CPA* and *AQ*. And both of their impact passed the significance test.

## 5 Robustness test

In order to avoid the randomness and non-scientificity of evaluation methods, explanatory variables and regression results, the robustness test was used for further validation and analysis. In this paper, the Models (1) and (2) were tested again by replacing the explanatory variable *AQ* with the audit cost *LnFee* (its natural logarithm) and the discretionary accrual |*Dacc*| (its absolute value) respectively. The robustness test results are shown in Table 5.

**Table 5:** Results of robustness test.

Variables	Model (1)		Model (2)	
	Columns(1)	Columns(2)	Columns(3)	Columns(4)
	LnFee	|Dacc|	LnFee	|Dacc|
H(X)	0.0852***(18.87)	0.0017**(2.71)	0.0852***(18.87)	0.0017**(2.71)
CR_4-Income_	4.8783**(3.14)	0.0139(0.07)		
CR_8-Income_	0.3156(0.20)	0.1587(0.81)		
HHI_Income_	-0.0042*(-2.07)	-3.08e-06(-0.01)		
CR_4-CPA_			3.1776(0.66)	-0.3260(-0.52)
CR_8-CPA_			8.5830(0.91)	-1.4370(-1.16)
HHI_CPA_			-0.0172(-1.09)	0.0020(0.98)
Income	0.7032***(34.30)	-0.0082***(-4.72)	0.7032***(34.30)	-0.0082***(-4.72)
CPA	-0.7975***(-30.07)	0.0059**(2.65)	-0.7975***(-30.07)	0.0059**(2.65)
Cons	yes	Yes	yes	yes
Industry	yes	Yes	yes	yes
Year	yes	Yes	yes	yes
R-squared	0.2409	0.0330	0.2409	0.0330
f	97.41	15.69	97.41	15.69
obs	14815	12648	14815	12648

The Columns (1) and (3) of Table 5 show the regression results of replacing *AQ* with *LnFee*. The regression coefficient of the information entropy value of KAM disclosures against *AQ* was 0.0852, a significant positive correlation, and this result held true at the 1% significance level. It suggested that *H*_1*a*_ held true, that is, KAM disclosures can improve audit quality, which was completely consistent with the main regression test results. The Columns (2) and (4) of Table 5 show the regression results of replacing *AQ* with |*Dacc*|. The regression coefficient of the information entropy value of KAM disclosures against *AQ* was 0.0017, a significant positive correlation, and this result held true at the 5% significance level. It suggested that *H*_1*a*_ held true, that is, KAM disclosures can improve audit quality, which was completely consistent with the main regression test results. To sum up, it passed the robustness test, which showed that the model setting was reasonable and scientific.

## 6 Further research

### 6.1 Impact of sector differences

In order to further study the impact of KAM disclosures in different sectors on audit quality, this paper adopted the information entropy value *H*(*X*)_*Index* of KAM disclosures based on the sector division system for testing. The details were as follows. Treat each sector as an independent system, and use Eq (1) to calculate the information entropy value of KAM disclosures in the audit reports within the system to replace the explanatory variable *H*(*X*) in Models (1) and (2). The regression analysis results are listed in Columns (1) and (4) of Table 6. The results showed that the impact of *H*(*X*)_*Index* on *AQ* was negative, but it failed the significance test, which requires more in-depth exploration and discussion. Although companies are listed in different sectors, the government’s regulatory requirements and intensity are almost equal. Thus, sector differences are not the main factor for CPAs and accounting firms to consider in KAM disclosures.

**Table 6:** Results of further research.

Variables	Model (1)			Model (2)		
	Columns (1)	Columns (2)	Columns (3)	Columns (4)	Columns (5)	Columns (6)
H(X)_Index	-0.0157(-1.26)			-0.0157(-1.26)		
H(X)_Industry		0.0097**(3.40)			0.0097**(3.40)	
H(X)_ACfirm			0.0252***(5.52)			0.0252***(5.52)
CR_4-Income_	32.5611**(2.81)	33.0086**(2.84)	31.6909**(2.73)			
CR_8-Income_	-3.1548(-0.28)	-4.0078(-0.35)	-3.8411(-0.33)			
HHI_Income_	-0.0337*(-2.07)	-0.0334*(-2.05)	-0.0332*(-2.03)			
CR_4-CPA_				54.9366(1.50)	53.7045(1.47)	56.7899(1.55)
CR_8-CPA_				170.6497*(2.35)	169.8735*(2.34)	178.0551*(2.45)
HHI_CPA_				-0.2839*(-2.32)	-0.2822*(-2.30)	-0.2940*(-2.40)
Income	-0.7712***(-6.07)	-0.7770***(-6.12)	-0.8907***(-6.80)	-0.7712***(-6.07)	-0.7770***(-6.12)	-0.8907***(-6.80)
CPA	0.6218***(3.52)	0.6236***(3.52)	0.7765***(4.19)	0.6218***(3.52)	0.6236***(3.52)	0.7765***(4.19)
Cons	yes	yes	Yes	yes	yes	yes
Industry	yes	yes	Yes	yes	yes	yes
Year	yes	yes	Yes	yes	yes	yes
Wald chi2	181.41	204.63	203.55	181.41	204.63	203.55
Wald chi2 p	0.0000	0.0000	0.0000	0.0000	0.0000	0.0000
Pseudo R2	0.0297	0.0318	0.0329	0.0297	0.0318	0.0329
obs	14797	14797	14797	14797	14797	14797

### 6.2 Impact of industry differences

Industry differences are one of the important factors for CPAs and accounting firms to consider in KAM disclosures, which is a universal consensus. In order to further study the impact of KAM disclosures in different industries on audit quality, this paper adopted the information entropy value *H*(*X*)_*Industry* of KAM disclosures based on the CSRC industry division system for testing. The details were as follows. Treat each industry as an independent system, and use Eq (1) to calculate the information entropy value of KAM disclosures in the audit reports within the system to replace the explanatory variable *H*(*X*) in Models (1) and (2). The regression analysis results are listed in Columns (2) and (5) of Table 6. The results showed that the regression coefficient of *H*(*X*)_*Industry* against *AQ* was 0.0097, a significant positive correlation, and this result held true at the 5% significance level. Thus, in the industry division system, there was a positive correlation between KAM disclosures and audit quality, and industry differences were a main factor for CPAs and accounting firms to consider in KAM disclosures.

### 6.3 Impact of accounting firm differences

Most studies believe that the Big Four accounting firms will be better than domestic accounting firms in communicating KAM disclosures. Accounting firms of different sizes and reputations differ in KAM intonation management strategies, and compared with the Non-Big Four accounting firms, the Big Four accounting firms had a stronger warning function of negative intonation and a stronger signal function of net positive intonation [30]. Based on the samples and statistical calculation, the number of key audit items disclosed in the audit reports issued by the Big 4 accounting firms was 2.148 per copy, the China’s top eight institutions were 2.043 units and the other China’s institutions were 1.995 units. In part, this suggested that larger, better-quality accounting firms were more likely to disclose more key audit matters and that the degree of homogeneity revealed by the Big 4 accounting firms was lower than that of China’s ones. In order to further study the impact of KAM disclosures in different accounting firms on audit quality, this paper adopted the information entropy value *H*(*X*)_*ACfirm* of KAM disclosures based on the accounting firm division system for testing. The details were as follows. Treat each accounting firm as an independent system, and use Eq (1) to calculate the information entropy value of KAM disclosures in the audit reports within the system to replace the explanatory variable *H*(*X*) in Models (1) and (2). The regression analysis results are listed in Columns (3) and (6) of Table 6. The results showed that the regression coefficient of *H*(*X*)_*ACfirm* against *AQ* was 0.0252, a significant positive correlation, and this result held true at the 1% significance level. Thus, in the accounting firm division system, there was a positive correlation between KAM disclosures and audit quality, and the Big 4 accounting firms were better than domestic accounting firms in the quality and the amount of information of KAM disclosures.

## 7 Research conclusion and enlightenment

Communication and disclosure of KAMs are the most important content of the new audit report reform. However, in view of the increasingly stereotype and homogeneity of KAM disclosures, it is particularly important and critical to timely test the actual effect of the new audit report reform and KAM disclosures. Based on the theory of information entropy, this paper selected the equation calculating the information entropy value of KAM disclosures in a single audit report, and took the KAM disclosures as a proxy indicator to study the relationship between KAM disclosures and audit quality. The results showed that KAM disclosures had a significant positive impact on audit quality. Previous studies used the logarithm of the number of KAM disclosures to study the impact on audit quality, while this paper used the information entropy value of KAM disclosures, which is of more scientific significance and is more worthy of our attention. The conclusions remained unchanged after the robustness test. Further research also found that there was a positive correlation between KAM disclosures and audit quality in the industry division system and the accounting firm division system; sector differences were the main factor for CPAs and accounting firms to consider in KAM disclosures; the Big 4 accounting firms were better than domestic accounting firms in the quality and the amount of information of KAM disclosures.

The conclusion of this paper shows that communication and disclosure of KAMs have a positive effect on audit quality improvement, and the impact mechanism of audit market concentration on KAM disclosures and audit quality has more possibilities. This provides empirical evidence and management enlightenment for auditor practice and the development of audit industry. From the perspective of the overall audit market, KAM disclosures indeed improve audit quality. In this environment, individual auditors should constantly improve their professional judgment ability, and positively view the effect of KAM disclosures on reducing the audit risk responsibility, otherwise they will be eliminated by the market due to their professional ability disadvantage. In China where the audit market concentration is low and the audit market is the competitive structure, the government should guide the accounting firms to undertake business according to their own scale, ability and expertise, promote the steady and orderly implementation of reform, promote the formation of a virtuous circle of competition, and stimulate the positive effect of audit market concentration on KAM disclosures and audit quality while reducing the negative impact.
